# A Rare Cause of Intestinal Obstruction in Infants: Ileum Duplication Cyst and Literature Review

**DOI:** 10.1155/2015/362478

**Published:** 2015-07-30

**Authors:** Mehmet Serif Arslan, Erol Basuguy, Hikmet Zeytun, Serkan Arslan, Bahattin Aydogdu, Mehmet Hanifi Okur, Mariah Ozkir, Ibrahim Ibiloglu, Ibrahim Uygun

**Affiliations:** ^1^Department of Pediatric Surgery, Dicle University Faculty of Medicine, 21280 Diyarbakir, Turkey; ^2^University of Kentucky College of Medicine, Lexington, KY 40506, USA; ^3^Department of Pathology, Dicle University Faculty of Medicine, 21280 Diyarbakir, Turkey

## Abstract

Cases of neonatal gastrointestinal system (GIS) obstruction are quite complex for pediatric surgery clinics. A rare cause of intestinal obstruction is the duplication cyst (DC). A three-day-old male patient presented at our clinic with a history of abdominal distension and bilious vomiting on the second day following birth. Although pathology had not yet been determined from observation and examination, surgery was performed when the patient could not tolerate oral feeding. An ileal DC forming an incomplete obstruction was observed. Ileoileal anastomosis was performed on the patient. Because DCs can present with different clinical symptoms, it is quite difficult to diagnose them in neonate patients. Lacking an imaging method that can provide an exact diagnosis, the diagnostic laparotomy is a suitable approach for both diagnosis and treatment to avoid delays in treatment.

## 1. Introduction

Duplication cysts (DCs), which may have either a spherical cystic or a tubular structure, are among the more rarely observed of congenital malformations of the gastrointestinal system (GIS). The first DC was described in 1733 by Calderin, followed by another in 1884 by Fitz. In 1937, Ladd defined DCs as having the following three properties: (a) the cyst is surrounded by smooth muscle, (b) the cyst must contain the GIS mucosa from which it takes its own origin, and (c) the cyst must have a wall in common with the anatomic region in which it is found [1]. DCs can be found anywhere in the GIS, from the mouth to the anus.

Though they are more often observed in the small intestine, DCs can also more rarely be rectal, duodenal, gastric, and thoracoabdominal. They have an incidence of 1/100,000 [2]. They become symptomatic depending on their localization. They generally evidence signs of compression. Complications include bleeding into the cyst, obstruction, perforation, fistulization, and, rarely, malignancy [3].

The literature demonstrates that delays may occur in treatment as DCs are rarely diagnosed in the neonatal period. We aim to present this rare case of ileal DC together with a literature review.

## 2. Case

**Figure 1:**
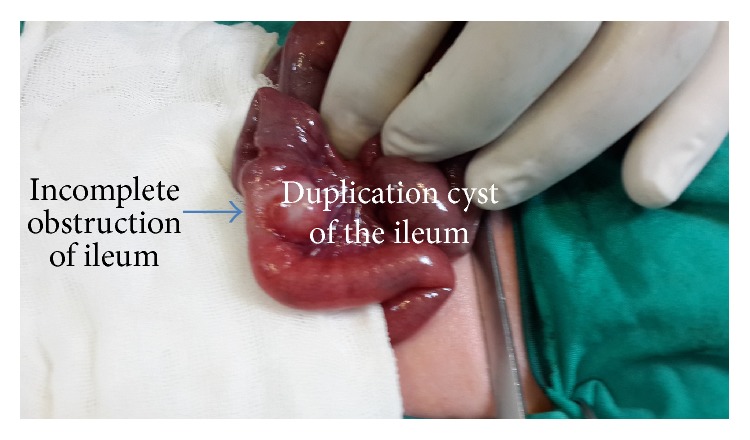
Intraoperative view: the ileal DC creating an incomplete obstruction that narrows the lumen.

**Figure 2:**
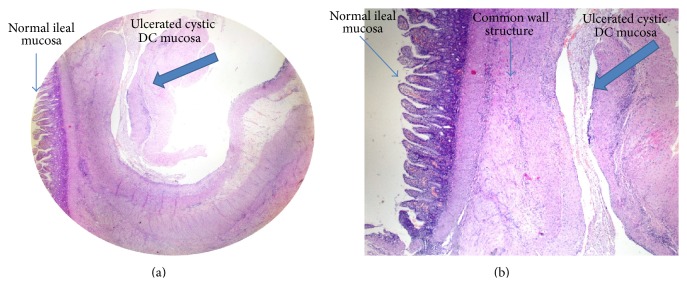
Pathologic examination of the patient. (a) Normal ileal mucosa (thin arrow) and ulcerated cystic DC mucosa (bold arrow) (HE; ×10). (b) View of the common wall structure between them. Normal ileal mucosa (thin arrow) and ulcerated cystic DC mucosa (bold arrow) (HE; ×40).

A three-day-old newborn male was admitted to the emergency department with a history of abdominal distension and bilious vomiting on the second day following birth. No pathology was visualized on either direct abdominal radiography or abdominal ultrasound. Routine tests and thyroid hormone levels were normal. Contrast enema radiography was also performed to examine the patient for neonatal congenital bowel pathology. No pathology was observed. After two days of normal oral feeding vomiting began again. Upon obtaining drainage containing fecalith from nasogastric intubation, together with an increase in distension, an emergency operation was planned. The exploration, performed on the fifth day following birth, revealed a cystic DC approximately 2 × 1 cm in size that was creating pressure in the lumen of the terminal ileum and causing distension in the proximal ileum (Figure 1). Ileal resection and ileoileal anastomosis were performed to address the luminal narrowing. On the third day of postoperative follow-up, residue-controlled oral feeding was resumed. Oral intake was normal. The patient was discharged on the fifth postoperative day. Postoperative follow-ups over the next two months were unproblematic. The pathologic analysis of the patient reported a cystic DC sharing a common wall structure with the ileum and advanced narrowing of the ileal lumen (Figure 2).

## 3. Discussion

DCs of the GIS are the result of one or more congenital anomalies of uncertain etiology. Theories regarding etiology include the persistence of fetal bowel diverticula, vacuolization, caudal duplication, and split notochord, but the theory that has gained the most acceptance has been split notochord syndrome [4]. DCs can be distinguished from other cystic lesions as they possess normal GIS epithelium. They are generally located on the mesenteric side and most often appear within the first year of life. Among adults they are mostly asymptomatic and may represent an incidental finding during other intra-abdominal procedures. Rarely, patients may present with acute abdominal symptoms. The classic scenario seen in children is partial intestinal obstruction and mobile abdominal mass. Depending on the localization of the cyst, rare complications such as obstruction, bleeding into the cyst, volvulus, cyst torsion, cystic rupture, infection of the cyst, urinary or biliary obstruction, or malignancy (3% sarcoma, lymphangiosarcoma) may arise [5]. DCs may be located anywhere in the GIS tract from the mouth to the anus. They are seen most often in the ileum (30%) and the ileocecal region (30%). Their frequency in other areas of the GIS has been reported as follows: 10% in the duodenum, 8% in the stomach, 8% in the jejunum, 7% in the colon, and 5% in the rectum [6].

Ultrasound and barium studies are among the most widely used imaging methods in the diagnosis of duplication cysts. CT and MRI scans are considered less necessary. Duplication cysts are defined on ultrasound by the presence of a hypoechoic outer muscular layer with an echogenic internal mucosal layer. Surgical excision is the recommended therapy for DCs [7].

DCs with different localizations and clinical symptoms may be encountered. Because there is no specific marker or imaging tool for them, we must include them among our differential diagnoses when patients become symptomatic. The diagnosis of DCs in the neonatal and infant periods can be quite difficult because patients present with nonspecific symptoms. Due to the difficulty of diagnosis in the period of infancy, fatal cases have been reported in the literature, diagnosed too late after clinical GIS obstruction [6]. We too were unable to visualize the DC in our patient before the operation. It was only during the laparotomy that we were able to diagnose and treat the condition. Only extremely large DCs might result in a prenatal diagnosis [8].

DCs may be of either a spherical cystic or a tubular type. In either case, the approach to treatment is the same: removal of the DC and anastomosis. We were unable to establish a preoperative diagnosis in our patient using ultrasound. We were able to diagnose the patient using explorative laparotomy, given the patient's prominent neonatal intestinal obstruction symptoms. The cystic-type DC we found in the ileum fit the classical definition and was exerting pressure on the lumen. We performed a resection and anastomosis as recommended by the literature, and we observed a complete recovery in the patient.

In conclusion, DC may be the etiology in neonatal congenital intestinal obstruction, albeit rarely. Delays may occur in diagnosis due to the incomplete obstruction which occurs in most cases. We are of the opinion that DC is an important provisional diagnosis for patients with recurrent complaints of intestinal obstruction.
